# The Effect of Clopidogrel and Ticagrelor on Human Adipose Mesenchymal Stem Cell Osteogenic Differentiation Potential: *In Vitro* Comparative Study

**DOI:** 10.1155/2024/2990670

**Published:** 2024-02-15

**Authors:** Sally S. Mohamed, Hala F. Zaki, Shereen N. Raafat

**Affiliations:** ^1^Pharmacology Department, Faculty of Dentistry, The British University in Egypt, Al Shorouk City, Egypt; ^2^Department of Pharmacology and Toxicology, Faculty of Pharmacy, Cairo University, Giza, Egypt; ^3^Dental Science Research Group, Health Research Centre of Excellence, The British University in Egypt, Al Shorouk City, Egypt

## Abstract

Ticagrelor (TICA) and clopidogrel (CLP) are extensively used antiplatelet drugs that act by antagonizing the P2Y12 receptors that are found on platelets in addition to bone cells. *Aim*. The purpose of this study was to investigate the effect of clopidogrel and ticagrelor on stem cells osteogenic differentiation *in vitro*. *Methods*. Human adipose-derived mesenchymal stem cells (hAd-MSCs) were divided into (1) control group, (2) osteogenic group (osteo group), (3) clopidogrel group (CLP group), and (4) ticagrelor group (TICA group). The osteogenic differentiation potential was determined by mineralization nodule formation using Alizarin Red S staining, measuring ALP enzyme activity by alkaline phosphatase assay. Quantitative determination for osteogenic markers included osteocalcin (OC); runt-related transcription factor 2 (RUNX2) performed using western blot; osteoprotegerin (OPG) using enzyme-linked immunosorbent assay (ELISA) and inflammatory markers; and tumor necrosis factor (TNF-*α*) and interleukin-6 (IL-6) measured using real-time polymerase chain reaction quantitative (RT-PCR) and ELISA. *Results*. In comparison to all study groups, the TICA group showed significant increase in the mineralized extracellular matrix, ALP enzyme activity, and bone markers expression as RUNX2 (*P* < 0.0001), OC, and OPG (*P* < 0.05). The expression of IL-6 and TNF-*α* was determined by RT-qPCR and ELISA techniques. TICA and CLP significantly decreased both markers compared to the control group. The TICA group showed statistically significant lower levels of both markers (*P* < 0.0001) than the CLP and control groups via the ELISA technique. *Conclusion*. TICA may possess a positive effect on hAd-MSCs osteogenic differentiation compared to CLP.

## 1. Introduction

Bone defects that cannot heal spontaneously are termed critical-size bone defects or large bone defects. These bone defects can emerge as a consequence of extensive trauma, multiple fractures, infection, tumor, or musculoskeletal disorders [1]. Bone healing process is an intricate physiological process that involves multiple cells and cell signaling molecules that interact at the site of fracture to repair bone tissue without scar formation [2]. In critical-size bone defects, the typical process of bone healing is insufficient to replenish the lost tissue.

Surgical procedures that involve bone grafting are widely used to address bone loss, utilizing autologous, allogeneic, or xenogeneic bone transplantation methods along with synthetic biomaterials. Autologous bone grafting is considered the most reliable approach for reconstructing extensive bone defects [3]. This process involves transplanting autologous tissue from healthy areas of the body; however, its application is limited by several factors, including the possible large bone volume needed, the associated pain, possible donor site morbidity, prolonged recovery period, and inadequate vascularization [4, 5]. Synthetic materials designed for bone grafting have been introduced to overcome the disadvantages of autologous bone transplantation of limited bone supply [6]. The primary costs associated with their fabrication prevent the wide application of these graft materials [7].

Adipose tissue-derived mesenchymal stem cells (Ad-MSCs) have the potential to self-renew, exhibit anti-inflammatory and immune-modulatory effects, and have the ability to differentiate into various cell types, including osteoblasts. Furthermore, these stem cells secrete molecules that can initiate or enhance tissue regeneration processes [4]. Adipose-derived stem cells (Ad-MSCs) can be obtained from adipose tissue by the liposuction process, which is considered a safe and minimally invasive procedure. Ad-MSCs have been extensively used in ex vivo experiments to test drugs or materials that may possess osteogenic potential, owing to their high osteoinductive potential and osteogenic activities, as well as their greater capacity for proliferation and formation of colonies when compared to stem cells from other sources, such as bone marrow MSCs [8, 9].

Clopidogrel is a thienopyridine that inhibits ADP-induced platelet activation via irreversible binding to platelet purinergic receptor P2Y12 [10]. Previous research has demonstrated that the P2Y12 receptor is also found on bone cells, including osteoblasts, osteoclasts, and osteocytes, in addition to platelets [11]. P2Y12 receptors' impact on bone cell biology and bone homeostasis has been the subject of conflicting research. Studies have claimed that P2Y12 receptors promote osteoblastogenesis and prevent the development of osteoclasts, while others have advocated maintaining osteoclast activity. Previous investigations have indicated the significance of ADP receptor P2Y12 in the formation and function of osteoclasts, particularly in models of pathological bone loss related to postmenopausal osteoporosis, rheumatoid arthritis, and bone metastases [12, 13]. Other studies also reported that these antiplatelet drugs decreased postsurgical bone loss, which may be attributed to their anti-inflammatory effects [14, 15].

Ticagrelor is a newer antiplatelet drug that acts by blocking the P2Y12 receptor and inhibiting cellular adenosine uptake through the equilibrative nucleoside transporter (ENT)-I. This mechanism results in increased extracellular adenosine levels. Ticagrelor inhibits osteoclast differentiation and increases extracellular adenosine levels, thereby stimulating adenosine A_2A_ receptors (A_2A_Rs) [10].

Accordingly, the aim of this study was to investigate the impact of both clopidogrel and ticagrelor on the osteogenic differentiation potential of human adipose-derived mesenchymal stem cells (hAd-MSCs) by detecting osteogenic-related markers including osteocalcin (OC), runt-related transcription factor 2 (RUNX2), and osteoprotegerin (OPG). In addition, the drugs' anti-inflammatory effect was assessed by measuring inflammatory-related markers such as tumor necrosis factor (TNF-*α*) and interleukin-6 (IL-6).

## 2. Materials and Methods

### 2.1. Materials Used and Resources

Ticagrelor (TICA), phosphate-buffered saline (PBS), collagenase I, bovine serum albumin (BSA), Dulbecco's modified Eagle medium (DMEM-glucose), Dulbecco's modified Eagle medium/Nutrient Mixture F12 (DMEM/F12) media, Oil-Red Stain, Alizarin Red Stain, Alcain blue stain, formaldehyde, and glacial acetic acid were purchased from Sigma-Aldrich (MO, USA). Clopidogrel (CLP) was from Sigma-Aldrich (Darmstadt, Germany). Fetal bovine serum (FBS) was from Gibco (NY, USA). Penicillin/streptomycin was from Thermo Fisher Life Scientific (NY, USA). Ascorbic acid-2-phosphate, dexamethasone, and para-nitrophenyl phosphate (p-NPP) were from Sigma-Aldrich (Steinheim, Germany). Primary antibodies of flow cytometry for CD90/PE, CD73/PE, CD105/FITC, CD34/PE, human leukocyte antigen (HLA)-DR/FITC, and CD45/FITC were obtained from Biosciences (CA, USA). Human Mesenchymal Stem Cell functional identification kit was from R&D systems (Minneapolis, USA). Beta-glycerophosphate was bought from Merck (Darmstadt, Germany). Prepared TM protein extraction kit (total protein) was from Bio-Rad (CA, USA). Bradford protein assay kit was from Bio Basic Inc. (Canada). For western blot, RUNX2 primary antibody was obtained from Cell Signaling Inc. (USA). Osteocalcin and *β*-actin primary antibodies were from Millipore Inc. (Germany). ELISA kits including osteoprotegerin (OPG), TNF-*α*, IL-6, and avidin-horseradish peroxidase (HRP) antibody were from Abcam (USA). MiScript Reverse Transcription Kit (QIAGEN) and a miScript SYBR Green PCR Kit were from Agilent Technologies (CA, USA).

#### 2.1.1. *In Vitro* Procedures

*(1) Ethical Statement*. The study protocol of this research has obtained approval from the Research Ethics Committee at Cairo University's Faculty of Pharmacy with the license number PT (2941, 3/2021).

### 2.2. *In Vitro* Stem Cell Isolation and Characterization

#### 2.2.1. Isolation and Culture of Human Adipose-Derived (hAd-) MSCs

The hAd-MSCs were derived from lipoaspirate obtained with informed consent from patients aged 28–40 years (*n* = 3) who were free from any underlying medical conditions. Lipoaspirate was collected by a plastic surgeon in two sterile plastic bottles (100 ml/bottle) that were immediately transferred to the laboratory under sterile conditions within 2 hours of the surgery. To isolate the stromal vascular fraction, the lipoaspirate was washed with sterile phosphate-buffered saline (PBS) multiple times. After that, the tissue underwent enzymatic digestion using 0.1% collagenase A in PBS containing 1% bovine serum albumin (BSA) for 60 minutes at 37°C with intermittent shaking. The digested lipoaspirate was then washed with Dulbecco's modified Eagle medium (DMEM) supplemented with 10% fetal bovine serum (FBS) and centrifuged at 600 g for 10 minutes. The resulted cell pellet was resuspended in PBS and filtered through a 200 mm mesh to eliminate blood and any residual tissue debris. The solution was centrifuged again, and the supernatant was resuspended in DMEM/F12 media containing 10% FBS and 1% streptomycin/penicillin. Isolated cells were seeded into four T-25 cell culture flasks with seeding density of 700,000 cells/flask. Next day, the suspended cells were removed and the media was changed every 3 days. Cell growth and morphology were observed under an inverted light microscope (Labomed, USA). After reaching 80% confluence, the cells were trypsinized and subcultured. Cells at passage 4 (P4) were used for all subsequent experiments [16].

#### 2.2.2. Characterization of the Ad-MSCs

For hAd-MSCs identification, immunophenotyping for mesenchymal stem cell surface markers and multilineage potential determination were performed.

*(1) Immunophenotyping Using Flow Cytometry*. The hAd-MSCs at fourth passage were trypsinized and centrifuged at 1200 rpm for 5 minutes. The resulting cell pellet was resuspended in PBS at a concentration of 1 × 10^6^ cells/mL. A volume of 100 *μ*L of the cell suspension was mixed with 10 *μ*L of a fluorescent-labeled monoclonal antibody (mAb) and incubated in a dark room at 37°C for 30 minutes. Following the incubation, the cells were washed with PBS containing 2% BSA and resuspended in PBS for immediate analysis using a flow cytometer (Cytofex, Beckman Coulter, USA). Different combinations of monoclonal antibodies (mAbs) were used, with each labeled with specific fluorochromes such as fluorescein isothiocyanate (FITC) and phycoerythrin (PE). The mAbs used against CD73/PE, CD105/FITC, CD90/PE, CD34/PE, CD45/FITC, and HLA-DR/FITC [17].

*(2) Determination of the hAd-MSCs Multilineage Differentiation Potential*. To evaluate the multilineage differentiation capacity of the isolated hAd-MSCs *in vitro*, cells from the fourth passage were cultured at a concentration of 5 × 10^4^ cells/mL in a 24-well plate. Multilineage differentiation was performed using the commercially available human mesenchymal stem cell functional identification kit, which included osteogenic, adipogenic, and chondrogenic differentiation media. The differentiation processes for each lineage were carried out following the instructions provided by the manufacturer. Noninduced hAd-MSCs cultured in complete growth medium (10% FBS-DMEM) served as control. After approximately 21 days of differentiation, adipogenesis was determined by the appearance of lipid droplets, which were assessed using Oil-Red staining. For osteogenic differentiation, staining by Alizarin Red-S was performed to detect extracellular matrix rich in calcium. Lastly, for chondrogenic differentiation, staining was performed using Alcian blue to confirm the production of sulfated proteoglycans [18].

### 2.3. Assessment of Clopidogrel (CLP) and Ticagrelor (TICA) Osteogenic Effect on hAd-MSCs

To determine CLP and TICA concentrations that should be used in our study, a viability assay (MTT assay) was performed using serial dilutions of both drugs (Supplementary Data A). The concentration of 1.8 *μ*M of TICA showed 100% cell viability (not cytotoxic) and was used for both drugs throughout the study experiments.

For induction of osteogenic differentiation, cells were seeded in a 6-well plate at a density of 30 × 10^4^ cells/well. After reaching 70% confluency, the medium was replaced with osteogenic differentiation medium, which composed of *α*-MEM, 10% FBS, dexamethasone 100 nM, ascorbic acid-2-phosphate 200 uM, and *β*-glycerophosphate 10 mM [19]. Cells were divided into 4 groups: cells cultured in osteogenic medium with 1.8 *μ*M clopidogrel [14] (CLP group), cells cultured in osteogenic medium with 1.8 *μ*M ticagrelor (TICA group), cells cultured in normal culture medium (DMEM) served as negative control (control group), and cells cultured in osteogenic medium only served as positive control (osteo group). Because all drugs were dissolved in dimethyl sulfoxide (DMSO), this solvent was added to the drugs and control media at a dilution of 1 : 10,000 [10]. Both osteogenic induction medium and basal culture medium were refreshed every three days for 3 weeks.

#### 2.3.1. Osteogenic Assays

*(1) Alizarin Red S Assay*. At the end of the osteogenic differentiation period, the presence of calcified mineralized nodules was detected and quantified among the study groups. The culture medium was removed, and the cells were fixed with a 10% formaldehyde solution at room temperature for 15 minutes. To eliminate any nonadherent cells, the plates were washed three times with phosphate-buffered saline (PBS). Subsequently, the cells were stained with a 20% Alizarin Red S solution (pH 4.2) for 30 minutes in the dark at room temperature. Following staining, the cells were rinsed three times with PBS to remove any excess stain, and they were imaged using an inverted light microscope (Labomed, USA). The Alizarin Red staining was then solubilized with a 10% glacial acetic acid solution, and the produced color was measured using a benchtop microplate reader at 405 nm [20]. All samples were performed in triplicate (*n* = 3), and the experiment was repeated three times.

*(2) Alkaline Phosphatase (ALP) Activity Assay*. To evaluate the alkaline phosphatase enzyme (ALP) activity, the monolayers of cells were rinsed twice with PBS, followed by an additional wash with 1 mL of alkaline phosphatase buffer (ALPB). Subsequently, each well was treated with 1 mL of ALPB and an equal volume of para-nitrophenylphosphate (p-NPP) that had been precooled to 4°C. Immediately after adding the p-NPP solution, 50 *μ*L aliquots were collected from each well and mixed with an equal volume of sodium hydroxide (NaOH) to stop the enzyme-substrate reaction. This sampling procedure was repeated every minute for a total of 10 minutes per well. In this assay, the ALP enzyme converts colorless p-NPP to yellow-color p-nitrophenolate (p-NP), which was measured by using a spectrophotometer at 405 nm. The rate of p-nitrophenolate accumulation (absorbance) was plotted for each well against time, and the initial rate of the reaction, indicating the reaction rate, was determined by calculating the slope of the curve in each experimental group [21]. All samples were performed in triplicate (*n* = 3), and the experiment was repeated three times.

#### 2.3.2. Determination of Osteogenic-Related Markers

*(1) Assessment of RUNX2 and OC Using Western Blot Analysis*. Bone markers, including RUNX2 and OC, were determined using western blot analysis. Total protein extraction was done using the ready-prepared TM protein extraction kit (total protein) according to the manufacturer's guidelines. The lysed pellets from all different groups were treated with sodium dodecyl sulfate (SDS) sample buffer to obtain the total cellular protein, which was subsequently heated at 95°C for 5 minutes at pH 6.8. The protein concentrations were determined using the Bradford Protein Assay Kit. 20 *μ*g of proteins were then separated on a 10% sodium dodecyl sulfate polyacrylamide gel electrophoresis (SDS-PAGE) and transferred onto a polyvinylidene difluoride membrane (PVDF). The blot was run for 7 min at 25 V to allow protein bands transfer from gel to membrane using Bio-Rad Trans-Blot Turbo. After blocking with PVDF Blocking Reagent containing 20 mM Tris pH 7.5, 150 mM NaCl, 3% BSA, and 0.1% Tween 20, the membranes were incubated overnight at 4°C with a 1 : 1000 dilution of RUNX2 primary antibody and a 1 : 500 dilution of OC primary antibody in tris-buffered saline with Tween 20 (TBST) buffer. Subsequently, the membranes were incubated with a horseradish peroxidase- (HRP-) conjugated secondary antibody solution (Goat anti-human IgG-HRP-1 mg Goat mAb) with a dilution of 1 : 5000 for 1 hour at room temperature. The blot was then washed multiple times with TBST before the protein bands were visualized on a photographic film (Sigma-Aldrich) [22]. Each sample was performed in triplicate (*n* = 3).

*(2) Determination of OPG Using ELISA Assay*. Osteoprotegerin (OPG), a marker for bone metabolism, was quantified using the enzyme-linked immunosorbent assay (ELISA) with a commercially available kit. The ELISA procedure was carried out following the manufacturer's instructions. The ELISA microplate was precoated with the primary antibody specific to each marker. After adding samples or standards, a biotinylated detection antibody specific for OPG was added. Avidin-Horseradish Peroxidase (HRP) conjugate was added successively to each well and incubated. Then, wells were washed and the substrate was added for 15 minutes. The enzyme-substrate reaction is terminated by the addition of sulfuric acid solution. The assay demonstrated a sensitivity of 4.69 pg/mL for detecting OPG; the intra-assay and interassay coefficients of variation for the ELISA tests were found to be below 10% and 12%, respectively. Absorbance measurements at a wavelength of 450 nm were performed using a UVN-340 ASYS Hitech GmbH microplate reader [23]. All samples were performed in triplicate (*n* = 3), and the experiment was repeated three times.

### 2.4. Assessment of Clopidogrel (CLP) and Ticagrelor (TICA) Anti-Inflammatory Effects on hAd-MSCs

#### 2.4.1. Determination of Proinflammatory Markers Using RT-qPCR

To assess the expression levels of the proinflammatory markers TNF-*α* and IL-6, cells were cultured with 1.8 *μ*M ticagrelor (TICA group) and 1.8 *μ*M clopidogrel (CLP group) for 7 days [24]. Cells in normal culture medium served as control. Cells were then collected (2.5 × 10^6^ cells/group) under standard conditions (37°C, 5% CO_2_), and total RNA extraction was carried out using the Trizol kit following the manufacturer's protocol. The extracted RNA concentration and purity were determined using a Nanodrop spectrophotometer (Thermo Fisher, USA). The ratio between sample absorbance at 260 nm and 280 nm was used to assess the purity of RNA. The RNA samples (1 *μ*g/sample) were retrotranscribed using the miScript Reverse Transcription Kit (QIAGEN) and a miScript SYBR Green PCR Kit. Real-time quantitative polymerase chain reaction (RT-qPCR) was performed using the QuantiTect® SYBR Green PCR Master Mix in final volume of 20 *μ*L per sample. The PCR thermal cycling parameters for mRNA were 2 min at 50°C, 30 s at 95°C, 35 cycles of 95°C for 10 s, and 60°C for 40 s. The obtained results were presented as fold change using the 2^−ΔΔCt^ method and normalized to the reference gene *β*-actin. A negative control that contains all the components of the reaction except for the cDNA was included in each PCR run to detect contamination or nonspecific amplification. The primer sequences used for amplification were assessed for specificity by NCBI Primer-BLAST and are provided in Table 1.

#### 2.4.2. Determination of Proinflammatory Markers Using ELISA

The proinflammatory markers, TNF-*α* and IL-6, were further quantified using the enzyme-linked immunosorbent assay (ELISA) with a commercially available kit. The assessment of proinflammatory markers was performed after incubating the cells with 1.8 *μ*M TICA and 1.8 *μ*M CLP for 7 days without osteogenic induction [25]. The ELISA procedure was carried out following the manufacturer's instructions as previously mentioned, using biotinylated detection antibody specific for TNF-*α* and IL-6. The assay demonstrated a sensitivity of 0.011 pg/mL for TNF-*α* and IL-6 levels. Absorbance measurements at 450 nm were performed using a UVN-340 ASYS Hitech GmbH microplate reader. All samples were performed in triplicate (*n* = 3), and the experiment was repeated three times.

### 2.5. Statistical Analysis

All experiments were conducted in triplicate (*n* = 3) and repeated in three independent runs. Statistical analysis was performed using the one-way ANOVA test, followed by pair-wise Tukey's post hoc comparisons using GraphPad Prism v8.1.0 software (GraphPad Software, San Diego, CA, USA). The results are expressed as the mean ± standard deviation (SD). *P* values less than 0.05 were considered statistically significant.

## 3. Results

### 3.1. Isolation of Human Adipose-Derived Mesenchymal Stem Cells (hAd-MSCs)

**Figure 1:**
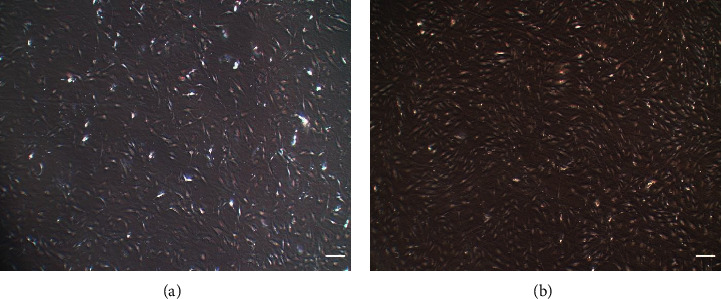
hAd-MSC morphology: (a) cells at passage 0 and (b) confluent cells at passage 4 (Mag. 40x). Scale bar: 250 *μ*m.

The isolated hAd-MSCs started to grow as adherent cells, attaining the characteristic fibroblast-like shape of the MSCs.  Figure 1(a) shows the hAd-MSCs five days after isolation (passage 0, P0). The cells continue to grow, attaining the same shape until passage 4 (P4) (Figure 1(b)). All experiments were done using cells in P4.

### 3.2. Flow Cytometry Characterization

**Figure 2:**
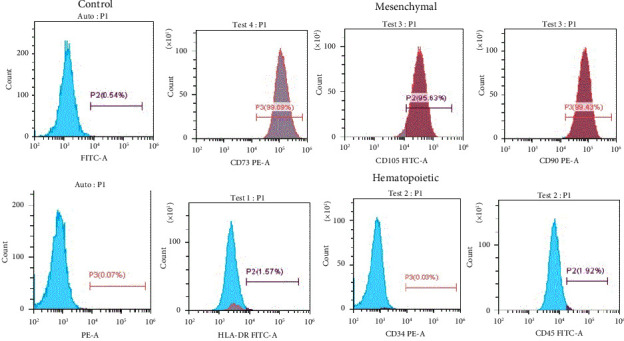
Flow cytometry analysis showing that 99.89% of the population of Ad-MSCs were CD73 positive, 90.63% were CD105 positive, and 99.43% were CD90 positive, while 98.34%, 99.97%, and 98.08% of these cells were negative for HLA-DR, CD34, and CD45, respectively.

The results of flow cytometry showed that the isolated cells were positive for MSC surface markers including CD73, CD105, and CD90 and negative for hematopoietic markers including HLA-DR, CD34, and CD45 (Figure 2). The percentages are presented relative to the proper expression of mesenchymal stem cell surface markers, indicating that isolated hAd-MSCs were a homogenous population of cells expressing MSC markers, while lacking hematopoietic markers fulfilling the criteria of MSC characterization.

### 3.3. Adipogenic, Osteogenic, and Chondrogenic Differentiation

**Figure 3:**
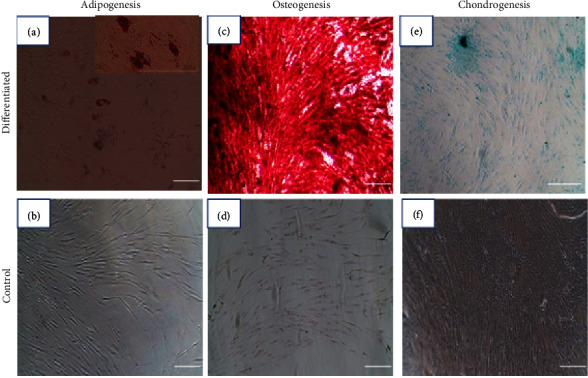
Multilineage differentiation of hAd-MSCs. (a) Adipogenesis was confirmed by oil droplets staining with Oil-Red compared to the control (b), (c) osteogenesis was confirmed by staining of mineralized nodules by Alizarin Red-S staining compared to the control (d), and (e) chondrogenesis was confirmed by staining proteoglycans with Alcian blue staining compared to the control (f). Magnification: 100×; scale bar: 250 *μ*m.

Multilineage differentiation of the isolated hAd-MSCs (adipogenic, osteogenic, and chondrogenic differentiation) was confirmed by special stains after 21 days of differentiation. Regarding adipogenic differentiation, droplets of oil were stained with Oil-Red staining (Figure 3(a)). For osteogenic differentiation, calcium-rich extracellular matrix started to accumulate and was stained with Alizarin-Red S staining (Figure 3(c)). Regarding chondrogenic differentiation, the produced sulfated proteoglycan was stained with Alcain blue (Figure 3(e)).

### 3.4. Osteogenic Assay Results

#### 3.4.1. Alizarin Red S Assay

**Figure 4:**
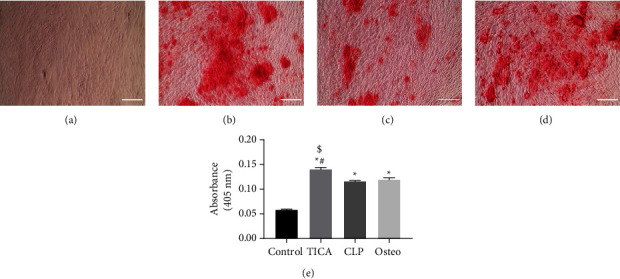
Calcium deposition, as a final product of the osteogenic differentiation process, was assessed using Alizarin Red S staining on day 21 (Mag. 100×). Scale bar: 250 *μ*m. (a) Control, (b) ticagrelor (TICA), (c) clopidogrel (CLP), (d) osteogenic medium, and (e) absorbance values (after stain solubilization) reported as mean ± SD of three independent experiments. ^*∗*^*P* significant difference of the respective group compared to the control. ^#^*P* significant difference of the respective group compared to the osteo group. ^$^*P* significant difference of the respective group compared to the CLP group. Data are expressed as mean ± SD of three independent experiments.

Osteogenic differentiation was confirmed by staining of calcium deposits (mineralized nodules) using Alizarin Red. After applying the osteogenic induction medium, the cells were observed regularly for morphological changes. The undifferentiated control hAd-MSCs were cultured in DMEM-F12 medium and showed no Alizarin Red labeling (Figure 4(a)). In the study groups, the calcium deposits started to accumulate at the end of the third week of osteogenic differentiation. The staining became denser, and multiple isolated mineralized extracellular nodules appeared (Figures 4(b)–4(d)). Statistical analysis showed significant increase in stain density between groups, which was measured by using a spectrophotometer after stain solubilization (Figure 4(e)). The results showed that the TICA group possessed the highest significant increase in absorbance (stain density) among the other study groups. The mean absorbance values were 0.0586 ± 0.0008, 0.140 ± 0.0032, 0.116 ± 0.0008, and 0.1197 ± 0.0035; *P* < 0.0001 in the control, TICA, CLP, and osteo group, respectively. Nonsignificant difference was observed in the CLP group compared to the osteo group (*P*=0.3880).

#### 3.4.2. Alkaline Phosphatase (ALP) Activity Assay

**Figure 5:**
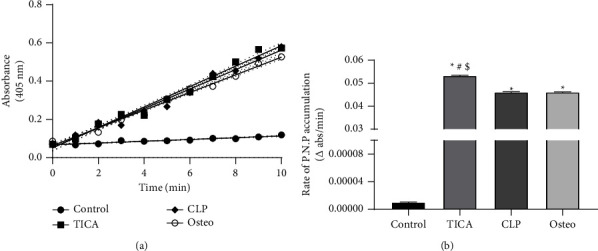
ALP activity after 21 days of osteogenic induction. (a) Representative kinetic profile of ALP assay demonstrating accumulation of the yellow p-nitrophenolate product over time among the different groups. (b) Rate of accumulation of the yellow p-NP was determined by calculating the slope of each reaction. The values reported are the means ± SD of three independent experiments. ^*∗*^*P* significant difference of the respective group compared to the control (cells in normal medium). ^#^*P* significant difference of the respective group compared to the osteo group (osteogenic medium). ^$^*P* significant difference of the respective group compared to the CLP group. *P* values <0.0001 are statistically significant. Data are expressed as mean ± SD of three independent experiments.

Alkaline phosphatase (ALP) enzyme activity was analyzed in the study groups after osteogenic differentiation. Results showed that, after culturing hAd-MSCs in osteogenic medium for 21 days, an increase in the ALP enzyme activity was observed in all study groups compared to the control group (Figure 5(a)). Statistical analysis was done by calculating the slope of each curve, demonstrating the initial rate of the reaction (p-NP accumulation). The control group possessed the lowest rate of accumulation, while the TICA group showed the highest rate. The ALP enzyme level increased significantly (*P* < 0.0001) in all groups in comparison with the control group as shown in Figure 5(b). Statistical analysis showed that ALP levels increased by 5 folds for the TICA group, 4.3 folds for CLP and 2.4 folds for the osteo group compared to the control group. CLP group revealed nonsignificant difference (*P*=0.9993) in comparison to the osteo group while the TICA group showed statistically significant increase in the ALP enzyme activity compared to all the study groups.

### 3.5. The Expression of Osteogenic-Related Genes OC and RUNX2 Using Western Blot

**Figure 6:**
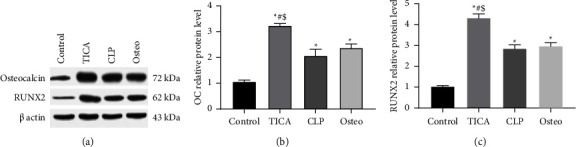
Western blot analyses of osteocalcin (OC) and RUNX2 in each group after 21 days of osteogenic induction. ^*∗*^*P* significant difference of the respective group compared to the control (cells in normal medium). ^#^*P* significant difference of the respective group compared to the osteo group (osteogenic medium). ^$^*P* significant difference of the respective group compared to the CLP group. Western blot results showed increased levels of OC and RUNX2 in the TICA group compared to other groups.

To assess the protein levels of OC and RUNX2 in the four experimental groups, western blot analysis was conducted. OC expression showed a statistically significant increase (*P* < 0.05) in the TICA group by 3 folds, 1.9 folds for the CLP group, and 2.2 folds for the osteo group in comparison with the control group (Figure 6(b)). The expression of RUNX2 was significantly increased (*P* < 0.0001) by 4 folds for the TICA group, 2.7 folds for the CLP group, and 2.8 folds for the osteo group when compared to the control group (Figure 6(c)). The CLP group showed nonsignificant difference in the expression of OC and RUNX2 (*P*=0.1451; *P*=0.7592), when compared to the osteo group. On the other hand, the TICA group revealed statistically significant increase in the expression of OC and RUNX2 compared to all the study groups. The full western blot images of Figure 6(a) were provided as Supplementary Data B.

### 3.6. The Expression of OPG Protein Levels in the hAD-MSCs Using ELISA

**Figure 7:**
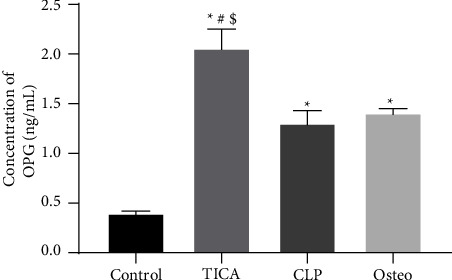
Osteoprotegerin (OPG) protein expression among the four groups after 21 days using ELISA. ^*∗*^*P* significant difference of the respective group compared to the control (cells in normal medium). ^#^*P* significant difference of the respective group compared to the osteo group (osteogenic medium). ^$^*P* significant difference of the respective group compared to the CLP group. *P* values <0.05 are statistically significant. The data are expressed as mean ± SD of three independent experiments.

A significant increase in OPG protein concentration was observed between groups with the highest percentage in the TICA group as shown in Figure 7. The OPG mean values were 0.395 ± 0.025, 2.055 ± 0.195, 1.3 ± 0.13, and 1.405 ± 0.045 (*P* < 0.05) in the control, TICA, CLP, and osteo groups, respectively. The CLP group showed no statistical significant difference (*P*=0.7149) compared to the osteo group; however, the TICA group demonstrated a statistical significant increase in sOPG level in comparison to all the study groups.

#### 3.6.1. The Expression of Proinflammatory Markers (TNF-*α* and IL-6) Levels in the hAD-MSCs

**Figure 8:**
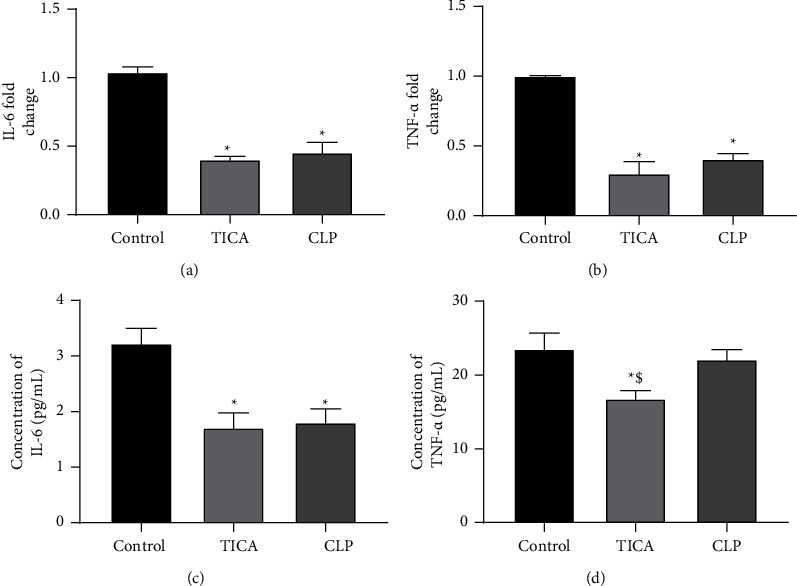
Proinflammatory markers expression. (a) The expression levels of IL-6 in the control, TICA, and CLP groups using RT-qPCR. (b) The expression levels of TNF-*α* in the control, TICA, and CLP groups using RT-qPCR. (c) IL-6 concentration in the control, TICA, and CLP groups via ELISA technique. (d) TNF-*α* concentration in the control, TICA, and CLP via ELISA technique. The data represent the mean ± SD from three independent experiments (*n* = 3). ^*∗*^*P* significant difference of the respective group compared to the control (cells in normal medium). ^$^*P* significant difference of the respective group compared to the CLP group. The data are expressed as mean ± SD of three independent experiments.

The mRNA expression of proinflammatory markers (IL-6 and TNF-*α*) was first evaluated among the three groups via RT-qPCR. The results showed that the expression of IL-6 was statistically significant lower in both TICA and CLP groups compared to the control group (Figure 8(a)), with mean values (1.039 ± 0.039, 0.399 ± 0.024, and 0.453 ± 0.074; *P* < 0.0001) in the control, TICA, and CLP groups, respectively. Similar results were observed for TNF-*α* expression; the relative expression of TNF-*α* was significantly lower in both the TICA and CLP groups in comparison with the control group (Figure 8(b)), with mean values (1.002 ± 0.002, 0.301 ± 0.087, and 0.406 ± 0.038; *P* < 0.0001) in the control, TICA, and CLP groups, respectively. Regarding both inflammatory markers expression, the TICA group showed lower expression compared to the CLP group; however, it was not statistically significant (*P* > 0.05). These results were confirmed by measuring these proinflammatory markers on protein levels among the study groups using ELISA assay, as previously mentioned. The concentration of TNF-*α* showed a significant decrease in the TICA group compared to the control and CLP groups, while the CLP group showed nonsignificant difference (*P*=0.548) compared to the control group (Figure 8(d)). The TNF-*α* level mean values are 23.55 ± 16.82, 23.55 ± 22.11, and 16.82 ± 22.11 (*P* < 0.05) in the control, TICA, and CLP groups, respectively. Similar results were observed regarding IL-6; significant decrease in concentration was observed in both the TICA and CLP groups compared to the control group (Figure 8(c)). Nevertheless, nonsignificant difference was observed between the two respective groups (*P*=0.9066).

## 4. Discussion

Healing large bone defects is considered a significant challenge in the medical field and can impede therapeutic interventions. While bone has inherent regenerative capabilities, pathologic or extensive fractures can hinder the natural healing process due to factors such as inadequate blood supply, infections in the bone or surrounding tissues, and other causes, leading to delayed repair [10]. Traditional bone grafting methods have limitations, necessitating the exploration of alternative approaches for addressing bone defects [26]. There are various surgical and pharmaceutical options available, but treating such conditions is challenging due to the complex processes involved in bone healing [10].

Limited research has been conducted on the osteogenic potential of antiplatelet drugs as TICA and CLP, and conflicting findings are found in the literature. Some studies have shown that CLP inhibits osteoblastic activity and bone regeneration [27], while others suggest that both TICA and CLP inhibit osteoclast differentiation and enhance osteoblastic activity *in vitro* [10–14]. In our study, we aimed to compare the osteogenic effects of CLP and TICA using a 1.8 *μ*M drug concentration, which showed no cytotoxicity on stem cells after performing a viability assay (MTT) using serial dilutions of both drugs. In addition, this dose was previously investigated by Comibra et al. [14] on human bone marrow-derived MSCs using CLP and reported that the drug had a positive effect on MSCs. As far as we know, this is the first study to examine and compare the osteogenic effects of both antiplatelet agents on hAd-MSCs.

In the present study, the osteogenic potential of TICA and CLP was determined on hAd-MSCs by several assays, including Alizarin Red S assay, ALP assay, bone markers expression measurement via ELISA, and western blot, in addition to detecting proinflammatory markers using RT-qPCR and ELISA technique.

Alizarin Red S is an *in vitro* assay used to detect osteogenic activity of cultured cells and quantify the produced calcified nodules. Our results stated that the amount of calcium-rich extracellular matrix was not significantly different between the CLP and osteo groups (cells cultured in osteogenic medium only), while the TICA group showed a significantly higher amount of calcium-rich extracellular matrix compared to both groups. Alizarin Red S assay results were further supported by the ALP assay which was employed to determine alkaline phosphatase activity, a key enzyme for osteoblast differentiation and mineralization, calorimetrically. Aligning with the Alizarin assay results, ALP activity showed the same pattern as the Alizarin assay results, and the TICA group showed an increase in enzyme activity compared to the CLP and osteo groups. Mediero et al. [10] investigated the local effects of TICA and CLP *in vitro* and *in vivo* on bone defects. The *in vitro* study showed that very low doses of both drugs (nM) inhibited osteoblast differentiation, while higher doses (*μ*M) had no negative effect on the osteogenic differentiation of the cells using the Alizarin Red S assay. The study also reported an increase in ALP protein expression in both groups *in vivo* using immunohistochemistry.

In contrast with our results, Syberg et al. [27] studied the role of CLP in bone homeostasis *in vitro* and *in vivo* using a 10 *μ*M dose of CLP on rat calvarial-derived osteoblast. The study showed a significant decrease in ALP activity *in vitro* using ALP colorimetric assay and a significant decrease in ALP expression *in vivo* using RT-qPCR. This is not consistent with the results obtained from our study, which showed that CLP did not possess a significant reduction in ALP activity in comparison to the osteo group; this could be attributed to the difference in the doses used in both studies.

In the current study, the expression of different osteogenic markers was measured, including osteocalcin (OC), a protein produced by osteoblasts [28], and RUNX2, a transcription factor essential for osteoblasts differentiation and proliferation, using western blot. In addition, osteoprotegerin (OPG), a soluble decoy receptor that inhibits osteoclast differentiation and activation [23], was assessed using the ELISA technique. Our findings revealed a significant increase in the expression levels of OC, RUNX2, and OPG in the TICA group compared to both the CLP and osteo groups. Our results agreed with Mediero et al. [10] who examined the expression of RUNX2 and OPG *in vitro* after the application of CLP and TICA. The study reported that TICA significantly increased RUNX2 expression, while CLP showed a slight but nonsignificant increase in the level of RUNX2. Regarding OPG expression, a significant increase was observed using both drugs; however, TICA showed a higher level than CLP. Syberg et al. [27] investigated the effect of CLP when applied to rat calvarial-derived osteoblasts using a 25 *μ*M dose. The study revealed that CLP decreased the expression of OC, while the expression of RUNX2 was unaffected. The differences in results among this study and our findings, as mentioned before, may originate from differences in the doses of the drug used.

Upon thorough research in the literature, previous clinical and experimental studies were found investigating the effect of CLP on bone metabolism; however, these studies showed controversies regarding the drug effect. Lillis et al. [29] investigated the effect of perioperative use of CLP orally on bone healing in rabbit calvarial bone defects. The study reported that continued perioperative oral use of CLP did not possess a negative bone effect but promoted fracture healing.

A cohort clinical study by Jørgensen et al. [12] investigated the relation between CLP administration and fracture incidence, in which all individuals had prescribed CLP during the years 1996–2008 in Denmark. The study results revealed a dose-dependent association between fractures risk and CLP usage. In comparison to controls, low-dose exposure was associated with a lower fracture risk, while higher exposure was associated with an increased risk of fracture. The study concluded that further research is required in order to investigate the potential effect of the antiplatelet drugs on bone metabolism *in vivo*.

Another recent study was conducted by Lv et al. [30] who collected data from the National Health and Nutrition Examination Survey (NHANES) and studied the association between osteoporosis or osteopenia using different antiplatelet drugs. The study showed that antiplatelet drugs including CLP were associated with osteoporosis in female subjects but not in males and recommended that further studies are needed to confirm the results.

The expression of proinflammatory markers such as IL-6 and TNF-*α* was determined in our study using RT-qPCR and ELISA. Our findings revealed that the expression of both proinflammatory markers decreased significantly in the TICA and CLP groups in comparison to the control group. TICA showed a slight lower expression than CLP, which was statistically nonsignificant. The results were further confirmed by measuring the aforementioned proinflammatory markers on protein level using an ELISA assay. The results showed that the TICA and CLP groups decreased both markers compared to the control group; moreover, the TICA group showed decreased levels of TNF-*α* compared to the CLP group. In line with our results, Coimbra et al. [31] investigated the anti-inflammatory effect of CLP and aspirin on periodontitis in a rat model and showed that both drugs significantly reduced the levels of IL-6 and TNF-*α*. A clinical study conducted by Thomas et al. [32] investigated the effects of TICA and CLP on immune responses. The results revealed that TICA significantly decreased IL-6 and TNF-*α*, while CLP significantly reduced TNF-*α* with a nonsignificant decrease in IL-6.

In the light of the current study results, using a low concentration of TICA on hAd-MSCs showed significant increase in the mineralized extracellular matrix, ALP enzyme activity, and bone markers expression as OC, RUNX2, and OPG, in addition to its anti-inflammatory effect through the reduction of IL-6 and TNF-*α* expression. In contrast, CLP did not show a significant positive impact on the bone metabolic markers; however, the drug possessed a significant anti-inflammatory effect using the same respective dose. The big controversies found in the literature about the CLP effect on bone metabolism and the results of our study suggest that the drug effect on bone metabolism is most probably dose dependent; the dose of CLP used in this study possessed no significant osteogenic potential.

In conclusion, the study showed that CLP did not possess osteogenic potential at the low dose used, compared to TICA which may possess a positive effect on hAd-MSC osteogenic differentiation. These results could pave the way for *in vivo* studies and further investigations on bone regeneration.

## Figures and Tables

**Table 1:** Sequence for primers used in RT-qPCR.

	Forward sequence	Reverse sequence	Gene accession number
TNF-*α*	ATGTTGTAGCAAACCCTCAAGC	AGGACCTGGGAGTAGATGAGG	NM_000594.4
IL-6	ACTCACCTCTTCAGAACGAATTG	CCATCTTTGGAAGGTTCAGGTTG	NM_000600.5
*β*-actin	TCCGTCGCCGGTCCACACCC	TCACCAACTGGGACGATATG	NM_006686.4

TNF-*α*, tumor necrosis factor-alpha; IL-6, interleukin-6.

## Data Availability

The data used to support the findings of this study are included within the article.

## Abbreviations

hAd-MSCs: Human adipose-derived mesenchymal stem cells
CLP: Clopidogrel
TICA: Ticagrelor
Osteo: Osteogenic
DMSO: Dimethyl sulfoxide
PBS: Phosphate-buffered saline
BSA: Bovine serum albumin
DMEM-glucose: Dulbecco's modified Eagle medium-glucose
DMEM/F12 media: Dulbecco's modified Eagle medium-F12
FBS: Fetal bovine serum
P-NPP: Para-nitrophenyl phosphate
OC: Osteocalcin
RUNX2: Runt-related transcription factor 2
OPG: Osteoprotegerin
ELISA: Enzyme-linked immunosorbent assay
TNF-*α*: Tumor necrosis factor
IL-6: Interleukin-6
ALP: Alkaline phosphatase
RT-PCR: Real-time polymerase chain reaction.

## Supplementary Materials

Supplementary Materials Supplementary Data A: a viability assay (MTT assay) was performed using serial dilutions of both drugs. Supplementary Data B: the full images of western blot for OC, RUNX2, and *β* actin (Figure 6(a)).
