# Supplementary material for: The Effect of Clopidogrel and Ticagrelor on Human Adipose Mesenchymal Stem Cell Osteogenic Differentiation Potential: In Vitro Comparative Study
Source: Adv Pharmacol Pharm Sci. 2024 Feb 15;2024:2990670. doi: 10.1155/2024/2990670 (PMC10883741; doi:10.1155/2024/2990670)
Supplement: Supplementary Materials — Supplementary Data A: a viability assay (MTT assay) was performed using serial dilutions of both drugs. Supplementary Data B: the full images of western blot for OC, RUNX2, and β actin (Figure 6(a)). [file 2990670.f1.zip › Supplementary Data A(MTT assay method).docx]

**Supplementary Data A**

Viability assay (MTT assay method)

**Method**

MTT assay was performed to compare the effect of Ticagrelor (TICA) and Clopidogrel (CLP) on hAd-MSCs viability after 3 days of culture. hAd-MSCs (10 x 10^3^ / well) were seeded in 96-well plates that were supplemented with complete culture medium. The plates were incubated at 37 ◦C in 5 % CO2 for 24 h. The original medium was removed and replaced with a medium containing serial dilutions of TICA and CLP (60–0.46875 μM). After 72 hours, the culture medium containing TICA and CLP was removed, and 1 mL of 3-(4,5-dimethylthiazol-2-yl)-2,5-diphenyltetrazolium bromide (MTT; MilliporeSigma) was added, followed by a 4-hour incubation period. The resultant violet formazan crystals, generated through the reduction of MTT by live cells, were then dissolved using dimethyl sulfoxide (MilliporeSigma). The resulting purple color was quantified using a microplate reader at a wavelength of 570 nm. Values obtained from each well were normalized against the control group (cells + medium only).

**Results**

Percentage of hAd-MSCs viability was determined using an MTT assay (Figure1). Data are presented after 72 hours of exposure of hAd-MSCs to different concentrations of TICA and CLP. Results were normalized to the control (cells + medium). Concentrations of 1.8 µM, 0.9 µM and 0.4 µM TICA showed 100% cell viability compared to other higher concentrations (p < 0.05), Whereas, CLP showed 99.8 % viability at concentration 15 µM and 100% cell viability using concentrations of 7.5 µM, 3.75 µM, 1.87 µM, 0.9 µM and 0.46 µM compared to other higher concentrations.

**Discussion**

The concentration of 1.8 µM was used in our study to compare between both drugs regarding osteogenic potential and anti-inflammatory effect. The used 1.8 μM concentration was also supported by a previous study conducted by Coimbra et al. 2015, who used CLP at 0.18 μM and 1.8 μM concentrations on human mesenchymal stem cells and reported that 1.8. μM concentration showed better cells proliferation *in vitro* (1).

A

B

**Figure 1. MTT assay results of TICA and CLP. A)** Cell viability % using serial dilutions of Ticagrelor (60–0.46875 μM). (B) Cell viability % using serial dilutions of Clopidogrel (60–0.46875 μM). Data are presented with absorbance values (570 nm) after 72 h of exposure of different concentrations of Ticagrelor and respectively, and their effect on cell viability. *p significant difference of this concentration compared with higher concentration of same respective drug. **p significant difference of this concentration compared to other concentrations of same respective drug.

1. Coimbra, L. S., Steffens, J. P., Alsadun, S., Albiero, M. L., Rossa, C., Pignolo, R. J., Spolidorio, L. C., & Graves, D. T. (2015). Clopidogrel enhances mesenchymal stem cell proliferation following periodontitis. Journal of Dental Research, 94(12), 1691–1697.
