# Supplementary material for: The Effect of Clopidogrel and Ticagrelor on Human Adipose Mesenchymal Stem Cell Osteogenic Differentiation Potential: In Vitro Comparative Study
Source: Adv Pharmacol Pharm Sci. 2024 Feb 15;2024:2990670. doi: 10.1155/2024/2990670 (PMC10883741; doi:10.1155/2024/2990670)
Supplement: Supplementary Materials — Supplementary Data A: a viability assay (MTT assay) was performed using serial dilutions of both drugs. Supplementary Data B: the full images of western blot for OC, RUNX2, and β actin (Figure 6(a)). [file 2990670.f1.zip › Supplementry Data B.docx]

**Supplementary Data B**


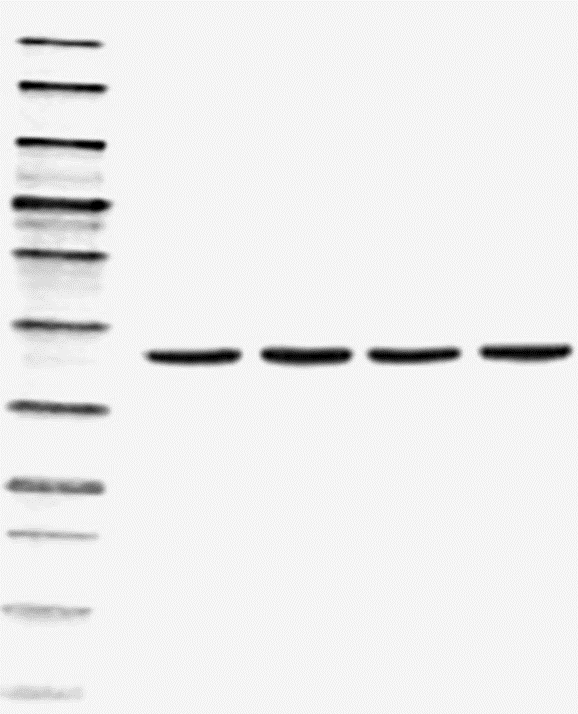

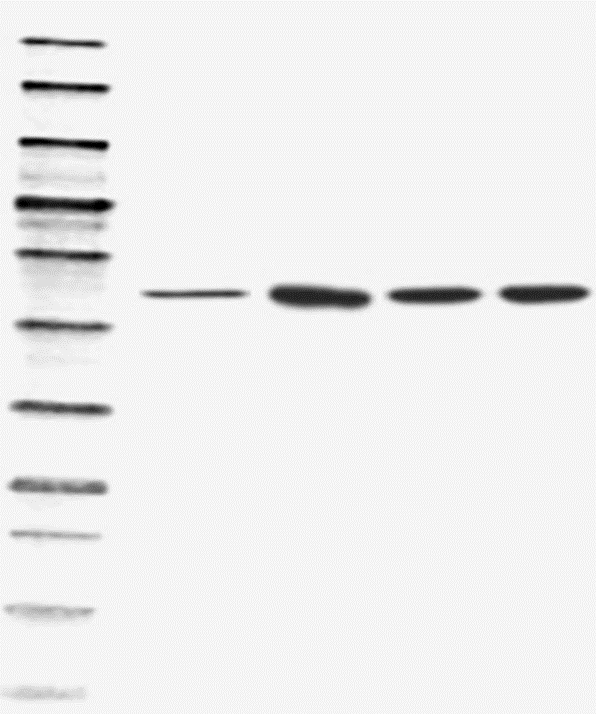

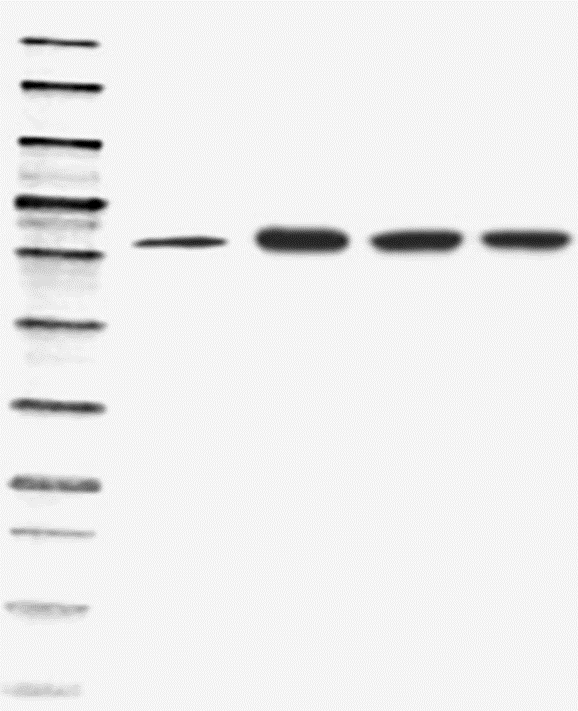


**C**

**B**

**A**

**Figure 1. Uncropped images of western blot of: A)** Osteocalcin at molecular weight 72 kDa, **B)** RUNX2 at molecular weight 62 kDa, **C)** β actin at molecular weight 43 kDa .
